# Interview with the Guest Editor—Ille C. Gebeshuber

**DOI:** 10.3390/biomimetics3020008

**Published:** 2018-04-17

**Authors:** Lidia Garcia-Campmany

**Affiliations:** *Biomimetics* Editorial Office, MDPI AG, St. Alban-Anlage 66, 4052 Basel, Switzerland; lidia.garcia@mdpi.com

## Abstract

Ille C. Gebeshuber is Professor of Physics at the Institute of Applied Physics at the Vienna University of Technology, Austria, where she graduated and completed her Ph.D. on technical biophysics of the inner ear in 1998. In 1999, she undertook postdoctoral training in scanning probe microscopy and biomimetics at the University of California, Santa Barbara, CA, USA, and soon after she returned to Austria to her home university to work on ion surface interactions, tribology and (bio-)nanotechnology. From 2009 to 2015, she joined the Institute of Microengineering and Nanoelectronics at the National University of Malaysia. During her expeditions, together with her students from cultural diverse backgrounds and expertise, she learned from the rainforest how nature develops well-adapted structures and materials, inspiring her to apply these principles to solve technological problems for humans to face global challenges in a safe and sustainable way. Her research focuses on nanotechnology and biomimetics, and takes a multidisciplinary approach, from biology and engineering to the fine arts and the social sciences. In 2017, she was elected Austrian of the Year in the “Research” category. We asked Ille about her career, her thoughts about the potential of biomimetic nanotechnology, and her experience during her editorship with *Biomimetics*.

**Figure:**
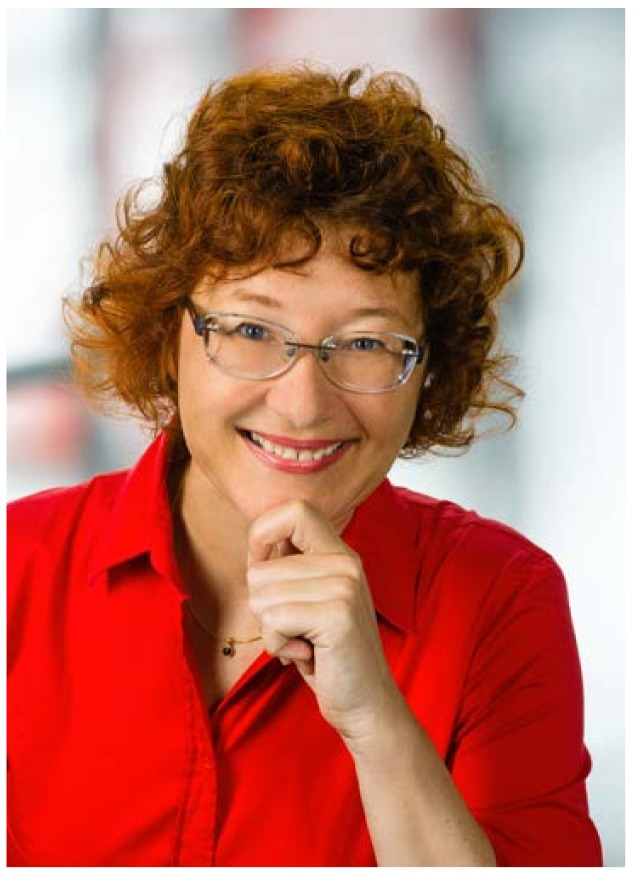
Image courtesy of Ille C. Gebeshuber (© Fotostudio Wilke, 1010 Vienna, Austria)

## How did you first become interested in biomimetic nanotechnology?

I have always been fascinated by life. Plants, animals and microorganisms make me happy. Specifically, when I found out about the amazing order of living matter on the micro- and nanoscale, I was absolutely fascinated and wanted to know more, understand more, transfer what I learn to engineering. I dealt with bacteria that make magnets with atomic precision, with the help of proteins—I immediately envisaged how we build our own data storage bits, with water-based chemistry, at ambient conditions. I learned about glass-making algae, of which tens of thousands of species exist—I wanted to have my beer glass grown in the river next to my cottage. Most important materials, structures and processes in living matter are based on functionalities on the nanoscale. And learning from them, via biomimetic principle transfer to engineering, to science, to the arts and to architecture, I see as important as beautiful.

## How relevant were those years you spent in Malaysia, and what was the impact on your life and scientific career?

We lived and worked for seven years in this amazing tropical country, where both modern cities and ancient beautiful rainforests can be found. I saw people who live in the jungle, get all they want and need from the jungle, and how their current life is impacted by modern society. On the other hand, I saw modern technology providing joy and fun to the young generation. I saw changes for the better, and for the worse. Most important for my approaches to science and engineering were my rainforest expeditions. I went to this strange, dangerous, beautiful place, being trained as an experimental physicist, as a nanotechnologist, as a tribologist (tribology is the science of friction, adhesion, lubrication and wear—whenever you have interacting parts in relative motion, you encounter tribological phenomena; be it the baby moving in her mother’s womb, the blinking of the eye, a car tire rolling on the street, or gliding, and writing with a chalk on the blackboard—tribology is everywhere). Being in this highly diverse jungle environment as a physicist, I noticed different things than the others, who were mainly biologists and tourists. I saw biological nanotechnology everywhere, safe, beautiful, sustainable biological nanotechnology. We drew various inspirations from these expeditions, and the essence of these seven years, and in a way also their climax, was my book “Where the machines are growing” [1], which provides ideas and approaches for positive technologies, technologies which are good for people and life in general. 

## What has been the most important lesson you have learned from nature?

The most important lesson I learned from Nature is the combination of beauty, expediency and diversity, which can be seen everywhere. In the tiniest lichen, in the interaction of a flower with a bee, in the coral reef ecosystem, in pioneer plants that colonize freshly sterilized volcanic grounds—these three aspects are always there. 

## What are the opportunities and challenges of biomimetic innovations?

Biomimetics is an amazing field. We have the whole living world to learn from, the most amazing molecules, animals, systems. The opportunities are vast, as are the challenges, especially given today’s way of education, focusing on specialization instead of on integration of knowledge across fields. Biomimetics is a field where you come only so far as a specialist. Opposed to science fiction novels, where the only boundary for you as a writer is your fantasy, in living entities, we see that it works. “It” might be weird things like navigation for thousands of miles in lobsters, or the bio-assisted accumulation of gold nanocrystals in yeast, or communication across biological kingdoms via horizontal gene transfer—we know that it works, we can analyze and understand it, and we can transfer it to our technological world. We know that it works. What we do not know, initially, is how and why. Here, the high integration of functionalities and also the multifunctionality that we often encounter in animated Nature can be a challenge for the researcher and developer. But what a wonderful challenge! Like a game, like a cypher manuscript, the answer is there, it exists, we just need to find it. Biomimetics can be described as a continuous pleasurable treasure hunt that is performed by smart people who know that there is a solution. 

## Your research is at the interface between biology, engineering, and the arts. How are these areas of knowledge integrated in your research? 

I think that the arts play an important role in bringing new ways of thinking to the general public. Explaining your thoughts in an artistic way is different than writing a scientific paper. Both are important, both are nice. With art, first fine airy ideas can be communicated, fires of enthusiasm can be lit, and then, with scientific papers, they can be materialized. I give you an example here: one of my great new loves is phytomining. Phytomining denotes mining for metals with plants. It is an amazing property that heavy metals, which are generally toxic, are not toxic to certain plants, and they take up the metals and incorporate them in their petals for example, which then taste awfully “metallic” for herbivores; this makes them turn to other plants to eat. Sometimes the plants take up so much heavy metals that it makes sense to burn them at the end of their growth season, and harvest the accumulated gold, or copper, or nickel, or rare earth metals, for use in our technologies. The plants accumulate the metals, and thereby also clean the soil from these metals, allowing agricultural plants—which are in many cases very sensitive to heavy metals and would not grow well—to grow there. The whole procedure of metal accumulation by plants is very complex, and needs loads of further research, especially if we want to have biomimetic principle transfer of the underlying principles to engineering; for example, for effective removal of heavy metals from industrial wastewaters, as we have in various Indian rivers, or recycling of electronic waste. Art can help here to make people and funding bodies aware of these plant superpowers. We currently grow metal accumulating plants in the entrance road of a major Austrian city, let them clean the polluted soil, then burn the plants at the end of their lifetime, extract the metals, and incorporate them in metallic pieces of art, with stickers “partly made by pollution, turned into resources”.

## What are you currently working on and where do you take inspiration from? 

I am currently working on safe biomimetic micro- and nanotechnologies. Positive technologies. Technologies that are not bad for people and the environment, but good. The three major pillars in my research are biomimetic materials, structures and processes. In the materials aspects, we establish new ways to obtain materials we need for our progress and development (like in phytomining). In the structures aspects, I like the motto “structure rather than material”: in various cases, smartly structured simple base materials can achieve the same or better functionalities than currently used advanced materials, with the additional benefit of being safe and beautiful. An example of this are the magical colors of certain butterflies that are produced by minuscule structures which play with the light, resulting in brilliant, non-bleaching coloration. Just recently, one of my Ph.D. students from the Fine Arts, Sigrid Zobl, developed a stamp onto which she transfers the color-giving structures of the morpho butterfly, yielding a device with which we can stamp colors without using paints or dies, just by transferring structures. This is just one example; in living Nature, we have an abundance of examples for this motto but we still need to learn a lot in this regard, because currently we think more in terms of materials than in terms of structures. The third pillar of my research is biomineralization processes, and their biomimetics. I am absolutely thrilled by the beautiful, functional forms of radiolaria, of bones of migratory birds, of the organic gemstones that are produced by certain plants, etc. The list is endless. There are more than 70 different biominerals, namely metals, alloys, ceramics, polymers and composites. They are produced with the help of proteins at ambient conditions. Examples are carbonates such as calcite (CaCO_3_) in mussels, phosphates such as hydroxyapatite in bones, oxides such as magnetite (Fe_3_O_4_) in magnetotactic bacteria, sulphates such as celestite (SrSO_4_) in radiolarians, sulphides such as pyrite (FeS_2_) in magnetotactic bacteria and native elements such as gold (Au) nanocrystals in yeast and sponges. We intend to transfer these smart, functional processes to engineering.

Where I take the inspiration from? By going through the world with open eyes, full of joy and curiosity. Every little patch where something grows is inspiring for me, every forest, every tree, every fir needle. Especially I love to get inspiration from organisms nobody loves. Mosquitoes, slime molds, leeches—by seeing them (also) as bionanotechnological wonders, the perception changes, and they become what they are—amazing creatures, beautiful, expedient, harmonious.

## What is your long-term research goal? 

My long-term research goal is to establish a way for people to live in harmony, in balance with living nature, by allowing all of us a good life, without harming the environment. I think the basis for such a life is deep respect for life. When we have this, everything else follows. 

## You have been elected Austrian of the Year 2017 in the “Research” category. How has your experience been interacting with the mainstream media?

I have always loved interacting with the mainstream media. My scientific career has been accompanied by radio interviews, TV appearances, as well as magazine and newspaper articles that have been written about me. I think people love my ability to communicate my research in a way that is interesting and that they can understand. It is one of my deep desires to have everybody understand what I do, why I do it, what it can do for society, and life in general, and what they themselves can do to contribute. I once gave a public lecture in a tram, to support free university education for our youth. I like to give lectures on nanorobots and biomineralization to pensioners. I go with kindergarden children to the butterfly house to visit the living jewels, and explain to them the bionanostructures that are responsible for the amazing coloration of certain butterflies, and then we go to the electron microscope at our university and play in the nanocosmos. Especially children from poor homes, underprivileged and without too much future, are my “target”. I think this comes from my own life story: I was the first in my family to ever enjoy higher education, and a legion of relatives and acquaintances followed my path. I have always been a pioneer. 

## You are active on social media and keep your personal website up-to-date. Where do you see the value for a scientist to have a public presence?

I want people to join in my joy, to continue what I do. In my research, which touches so many different aspects, goes into many different directions, with the common goal of developing positive technologies, I can only start to write the first lines. Finishing the book is the task of my fellow researchers and the ones who come after me. And then there is another, non-scientific aspect. I had two heartwarming encounters after having published my book. The first one was with a 96-year-old man. His wife had died recently, he had given up. He wanted to starve to death. He was walking over the main square in his little hometown, Steyr, and passed by the bookstore. He used to be an airplane engineer, and the title of my book caught his attention. And imagine what happened: he bought it, sat down in the local coffee house, read the first 37 pages in one sweep, got back his joy for life and is still happy and healthy, years later. I cried tears of joy when we finally met, and he read me a long letter of dedication, and gave me a huge beautiful bunch of flowers. Also, the next story is around flowers, and my book, and actually about people and their relations. One day, the florist called and brought me a beautiful bouquet, with an attached card. I met the woman who sent me the goodies, and she told me that she had heard one of my radio shows, a seven-part series on colors, and then, when her husband was diagnosed with cancer, my book was the thing that kept her up and going. She read it in this hard time, and it gave her hope, and helped her to endure. Actually, this is why we write—we can reach lives, we can help, we can change things for the better. Lovely. 

## You have engaged in several outreach initiatives. What is your advice to young, budding scientists?

My advice to young, budding scientists is to do what they really like to do. Then opportunities will pop up everywhere. They also need to be cautious—the current science system allows only a handful to stay in science, to get a permanent job. They should not forget about having a wife, a husband, kids, about their private life. 

## You have served as an Editorial Board member of *Biomimetics* since its official launch in 2016. How do you see the journal providing resources to the community? 

I think that *Biomimetics* is a great journal that serves the community well. I like Open Access, because in this case also people from outside science, actually people from everywhere, can access papers written by the best of the best for free, anytime. This may help foster a culture of greater scientific education and literacy. *Biomimetics* has managed to attract important papers from scientists who shape the field, and its Editorial Board reflects this top quality. I have been guest editor for various MDPI journals, and also admire the quality and professionalism of the administrative support. I thank you all and look forward to the future!
